# Not all butterflies are monarchs: orientation using daytime celestial cues but not magnetic cues in European migratory red admirals (*Vanessa atalanta*)

**DOI:** 10.1098/rsif.2026.0537

**Published:** 2026-10-21

**Authors:** Aleksandr Pakhomov, Anatoly Shapoval, Nazar Shapoval, Dmitry Kishkinev

**Affiliations:** ^1^ School of Life Sciences, Keele University, Staffordshire, UK; ^2^ Biological Station Rybachy, Zoological Institute RAS, Rybachy, Russia; ^3^ Department of Karyosystematics, Zoological Institute RAS, Saint Petersburg, Russia

**Keywords:** red admiral, *Vanessa atalanta*, flight simulator, orientation behaviour, magnetic compass, sun compass, clock-shift, insect migration

## Abstract

Seasonal migration in animals is a widespread and complex phenomenon, yet the mechanisms underlying orientation and navigation remain poorly understood in many taxa. In Lepidoptera, most insights come from monarch butterflies (*Danaus plexippus*) and Australian bogong moths (*Agrotis infusa*), which use both visual and geomagnetic cues for orientation. However, these species undertake multigenerational round trips between breeding grounds and specific non-breeding areas and are, therefore, not representative of most other lepidopteran migrants, for which the sensory mechanisms guiding orientation remain largely unexplored. Here, we report findings from a 2-year study investigating orientation mechanisms in the red admiral (*Vanessa atalanta*), a European migratory butterfly, under controlled light and magnetic conditions. Our results demonstrate that (i) red admirals primarily rely on solar cues for selecting their migratory direction; (ii) they show no response to clock-shift manipulation; and (iii) there is no evidence of magnetic compass use. These findings contrast with monarch butterflies, which rely on both a time-compensated sun compass and a light-dependent magnetic compass, and reveal interspecific variation in compass use among diurnal Lepidoptera. This underscores the need to expand orientation research beyond traditional model systems to better capture the diversity of migratory strategies and orientation mechanisms across Lepidoptera.

## 1. Introduction

Each year, billions of animals undertake seasonal migrations, embarking on journeys that span vast distances and encompass a wide range of environments. These remarkable movements, observed across diverse taxa (from tiny insects to large mammals), represent one of nature's most compelling phenomena. Understanding how animals accomplish these feats has long been a central focus of behavioural ecology, particularly the mechanisms underlying orientation (the ability to determine and maintain direction of locomotion using compasses, direction finding) and navigation (the capacity to determine one's position relative to a goal without direct sensory contact, position-finding). To date, our knowledge of these mechanisms is largely based on research on a limited number of model species from different vertebrate taxa. For example, birds are known to rely on three independent orientation mechanisms: a light-dependent inclination magnetic compass [1], a time-independent star compass [2,3] and a time-compensated sun compass [4,5] (a similar sun compass has been documented in fish [6–8]). Furthermore, birds can derive positional information from the Earth's magnetic field [9,10]. Geomagnetic cues for orientation and/or navigation have likewise been demonstrated in other migratory vertebrates, including sea turtles, salmon, eels and amphibians [11–17].

By contrast, there remains a significant knowledge gap concerning the orientation mechanisms of migratory Lepidoptera. Most of what we know about the compass systems of these insects comes from studies on just two species: the North American monarch butterfly (*Danaus plexippus*) and the Australian bogong moth (*Agrotis infusa*). Monarch butterflies are diurnal migrants that each autumn undertake an approximately 4000 km journey from the US and southern Canada to overwinter (hibernate) in the oyamel fir forests of central Mexico [18]. They rely primarily on solar information using a time-compensated sun compass for orientation [19,20], similar to migratory hoverflies in Europe [21]. Studies suggest that monarchs possess a light-dependent inclination magnetic compass similar to the avian magnetic compass, which requires UV-A/blue light [22–24]. However, in monarchs, the magnetic compass appears to function mainly as a backup system: individuals subjected to clock-shift treatments maintain their predicted time-compensated orientation despite exposure to the Earth's magnetic field and may rely on magnetic cues only when celestial cues are absent [25]. Another well-studied lepidopteran migrant, the Australian bogong moth, undertakes an approximately 1000 km journey each austral spring from various regions in southeast Australia to mountain caves in the alpine areas of New South Wales and Victoria, where they aestivate during the dry season [26,27]. These moths use information from the geomagnetic field together with visual landmarks to steer their nocturnal migratory flights [28]. A more recent study shows that bogong moths can also use stellar cues to choose a population-specific migratory direction [29].

Monarch butterflies and bogong moths share a distinctive migratory strategy: they undertake regular annual multigenerational round trips, with the *same* individuals travelling from breeding grounds to highly restricted non-breeding sites and then initiating the return migration in the following season [30]. Such a migratory strategy is not widespread among most migratory Lepidoptera [25]. Instead, many species exhibit continuous, year-round movements without entering diapause. These migrations typically span multiple generations, with different generations completing different parts of the annual cycle, as seen in species such as the painted lady (*Vanessa cardui*) and the red admiral (*Vanessa atalanta*) in Europe. Each year, painted lady butterflies perform an incredible, multigenerational annual round trip of approximately 12 000–14 000 km between tropical West Africa and Europe [31–34] and complete a trans-oceanic flight exceeding 4000 km between Africa and South America [35], strongly suggesting well-developed compass systems are at play. Although this species has recently been widely studied and is considered a popular model in butterfly migration research [34,36–38], only one study has attempted to investigate their orientation abilities under laboratory-controlled conditions [39]. The results of this study indicate that laboratory-reared painted ladies, despite showing highly significant southward orientation when tested outdoors with access to solar cues, exhibited no evidence of magnetic orientation or time compensation for the sun's movement, unlike monarch butterflies.

The red admiral, another well-known European migratory butterfly, undertakes a fairly regular migration from northern Europe to the Mediterranean region in autumn [40–44]. After reaching the Mediterranean, they overwinter there, typically mating, laying eggs on host plants such as nettles (*Urtica* spp.; [42]), pellitory-of-the-wall (*Parietaria judaica*) and hops (*Humulus lupulus*) and then dying. In spring, a new generation emerges and begins migrating northward, with individuals laying eggs on host plants along the way. One or two additional summer generations subsequently continue the recolonization of northern Europe [42]. In autumn, the main wave of red admirals initiates southward migration towards wintering sites in southern Europe and North Africa. Owing to this full annual cycle, red admirals show two peaks of abundance across Europe, with their numbers in autumn being dramatically higher than in spring [42,44,45].

Despite the migration ecology of red admirals making them an ideal model for studying orientation mechanisms in migratory butterflies, virtually nothing is known about the directional cues they use as orientation references during seasonal migrations. To address this knowledge gap, we conducted orientation experiments on red admirals under different light and magnetic field conditions during both spring and autumn migrations. We hypothesized that red admirals, like other diurnal insect migrants such as monarch butterflies and hoverflies, primarily rely on visual cues (the sun compass) and compensate for the sun's daily movement using internal clocks. Further, we investigated whether they are able to choose an appropriate migratory direction when access to the sun and solar cues is restricted and the geomagnetic field is the only available directional cue.

## 2. Material and methods

### 2.1. Butterfly capture and husbandry

Red admirals of both sexes were collected during spring (May 2024) and autumn (August to September 2023–2024) migratory seasons at the ‘Fringilla’ field station (Kaliningrad region, Russia, figure 1A) in large ‘Rybachy-type’ traps (length: 70 m, width: 30 m, height: 15 m) typically used for migratory songbirds (supplementary material, figure S1A; see details in [43,44]). Upon capture, butterflies were housed indoors in a windowless laboratory chamber, in large mesh cages (47 × 47 × 47 cm). They were maintained under controlled conditions, including a constant temperature (21–22°C during the light phase and 15–17°C during the dark phase), 75–80% relative humidity and an artificial photoperiod mimicking natural day–night cycles (spring L : D approx. 16 : 8–17 : 7; autumn L : D approx. 16 : 8–12 : 12, the photoperiod changed gradually throughout the study in parallel with the natural seasonal changes in day length). REMEZ SunLike LED bulbs (RemiLicht GmbH, Germany) served as a main light source, and ZooDa Bird Compact bulbs (ZooDa TM, Russia; see spectra in supplementary material, figure S2) served as a UV source; both were automatically switched on/off at local sunrise/sunset by a Shelly 4Pro smart dimmer (Allterco Robotics, Bulgaria). The butterflies were fed 10% honey solution (in water) and housed under these conditions for at least 3–5 days before testing to recover from capture-induced stress. After orientation tests, all red admirals were released in good body condition, with no signs of damage to wing structure or scales, and well before the migration of their conspecifics had finished. Butterflies retained normal flight ability and showed no visible structural damage that would be likely to impair survival or flight performance after release.

**Figure 1.**
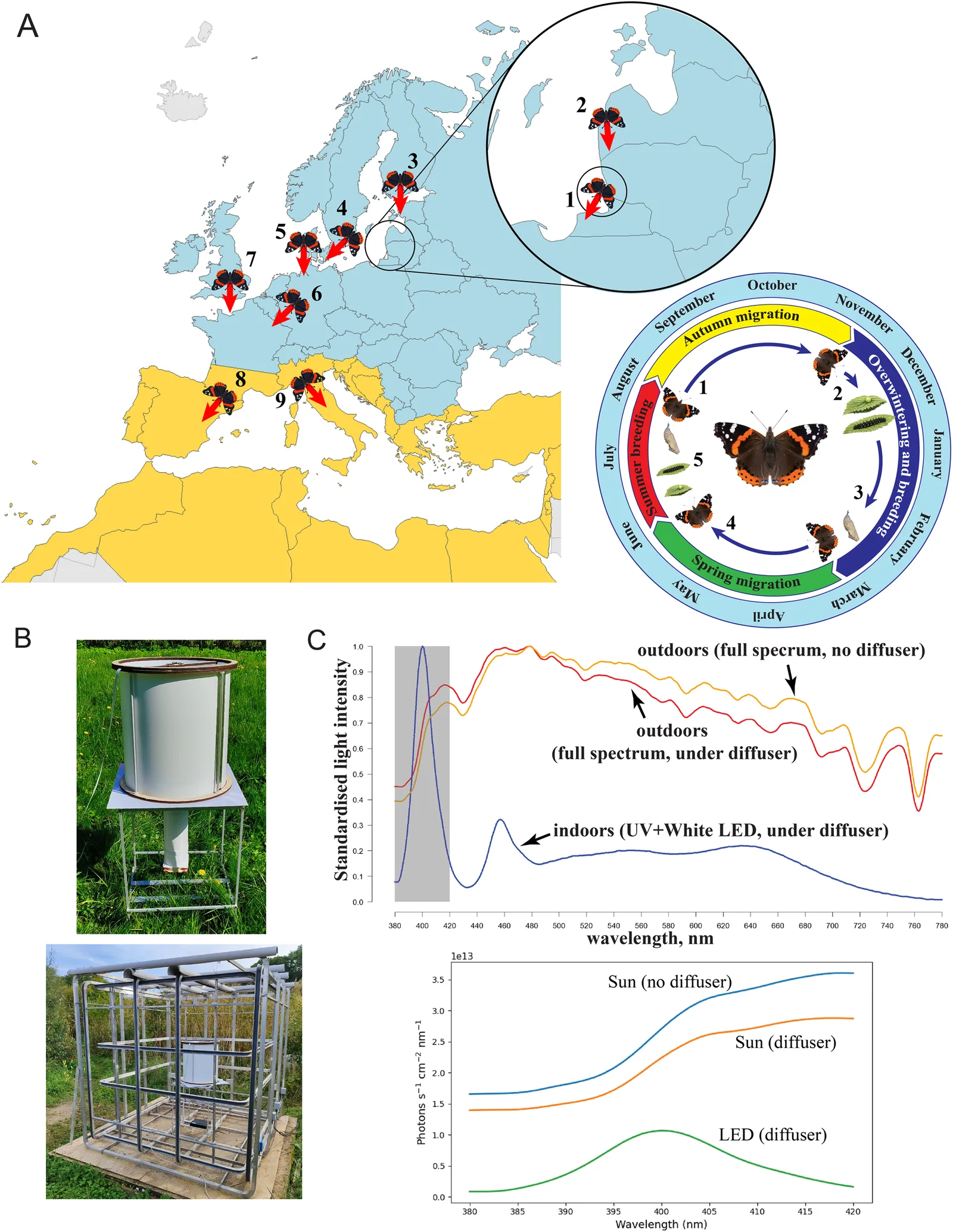
Migration of red admirals in Europe and experimental methods for studying orientation mechanisms in migratory butterflies. (A) Map showing the distribution of red admirals, which are widespread and abundant across Europe and their likely migratory destination (yellow: Mediterranean region). Admiral icons and arrows represent the directions of red admiral migration during the autumn season, based on field studies using visual observations and orientation experiments: (1) study site: Russia, [44] (flight simulator); (2) Latvia, [44] (visual); (3) Finland, [46] (visual); (4) Sweden, [47] (circular cages), [48] (visual); (5) Denmark, [49] (visual); (6) Germany, [50] (visual); (7) UK, [51] (visual); (8) Spain, [42] (visual); (9) Italy, [52] (visual). The circular diagram on the right represents the life cycle of red admirals. Summer generations typically begin their autumn migration from northern Europe towards the Mediterranean region in August (1). After reaching the wintering areas, adults usually mate, lay eggs and die (2). A new generation develops slowly during the winter (3), and after emergence, adults initiate spring migration towards central and northern Europe (4), where they reproduce. The resulting summer generations (5) then begin the autumn migration again. (B) Experimental set-ups for outdoor experiments on red admirals under full-spectrum light: in the natural magnetic field (NMF) (up) and 120° clockwise (CW)-rotated magnetic field (MF) (down). (C) Spectrum and irradiance curve of lighting conditions during flight-simulator tests. Light measurements were taken inside the flight simulator, at the position of the heads of tethered red admirals. Upper panel: standardized light intensity under different experimental conditions: outdoors with a view of the sun (total intensity: approx. 12.4 × 10¹⁹ photons s⁻¹ m⁻²), outdoors under a diffuser (total intensity: approx. 2.4 × 10¹⁹ photons s⁻¹ m⁻²) and indoors under LED artificial light (total intensity: approx. 8 × 10^18^ photons s^−1^ m^−2^). All spectra were standardized by taking the maximum value within the measured wavelength interval as 1.0, similar to [53]. Lower panel: irradiance curves for the light conditions used in our experiments across 380–420 nm, a spectral range critical for magnetic orientation in migratory monarch butterflies (sun outdoors: 2.7 × 10¹³; diffuser outdoors: 2.1 × 10¹³; LED indoors: 5.2 × 10¹² photons s⁻¹ cm⁻² nm⁻¹).

### 2.2. Flight simulator

All behavioural experiments were performed in flight simulators modified from [20], which allow butterfly flight tracks to be obtained and analysed for orientation behaviour (figure 1B; supplementary material, figure S1B,C). The flight simulator was constructed from non-magnetic materials (polyvinyl chloride (PVC), aluminium and polyethylene terephthalate glycol (PETG)) and comprised two main components:

1) **Flight chamber**. The flight chamber consisted of a white plastic cylinder (45 cm diameter, 50 cm height) mounted vertically on a plastic/aluminium table. A 3D-printed plastic pipe (120 mm in diameter) with several hundred 3 mm holes was placed at the table's centre to produce laminar airflow (less than or equal to 1 m s⁻¹). Airflow was generated by a pulse width modulation (PWM)-controlled computer fan and was used to stimulate active flight in tethered butterflies [20]. Butterflies were attached to a tethering apparatus consisting of a plastic bar mounted across the top of the cylinder. A 15 cm fine tungsten rod (0.5 mm diameter) was connected to the bar as the central shaft. A small plastic tube (20 mm long, 2 mm diameter, with a 0.5 mm hole) was fixed to the distal end of the rod to allow quick and secure attachment of butterflies. The upper edges of the white cylinder restricted the butterflies’ visual access to the sky to approximately 110°.
2) **Direction-recording system**. Two miniature video cameras (Waveshare Electronics, model H) were mounted symmetrically around the tungsten rod, with one camera recording the butterflies’ behaviour from above. The camera was connected to a Raspberry Pi 4 single-board computer, housed in a grounded aluminium enclosure together with a power bank. This system was used for real-time observation of red admiral flight behaviour and for storing raw video recordings (format: .h264, resolution: 1080p, frame rate: 25 fps) from each test for subsequent analysis.

### 2.3. Experimental procedures

#### 2.3.1. Indoor experimental procedures and light conditions

All flight-simulator experiments under artificial light were conducted in a purpose-built non-magnetic laboratory house with controlled temperature (21–22°C). The house comprised two rooms: a larger primary experimental chamber (4 × 4 × 4 m) and a smaller equipment room. The experimental chamber walls were plated with aluminium and grounded to form a Faraday cage. A double-wrapped, three-dimensional Merritt four-coil system (‘magnetic coils’ hereafter) was positioned in the chamber to allow manipulation of the magnetic field during experiments (supplementary material, figure S1D). This Merritt coil system generates a magnetic field with greater than or equal to 99% homogeneity within a volume of approximately 110 × 110 × 110 cm, at the centre of which we placed the flight simulator. Each of the three coil axes was powered by a separate BOP 50-4M power supply (Kepco Inc., USA), allowing precise control of the field intensity and direction. Magnetic field parameters were checked in the centre of the flight simulator using an FVM400 portable magnetometer (Meda Inc., USA) before tests. Professional LED strips (Arlight, Russia) served as the artificial light source (395 nm UV LEDs + white LEDs (5000 K, CRI = 95–98)). According to previous studies on monarch butterflies, their magnetic compass requires exposure to specific UV/blue light (380–420 nm) at a sufficient irradiance level (greater than 10^11^ photons s^−1^ cm^−2^ nm^−1^; [22–24]). The light intensity of our LED light source met these requirements: total intensity: approximately 8 × 10^18^ photons s^−1^ m^−2^, 380–420 nm: approximately 5 × 10^12^ photons s^−1^ cm^−2^ nm^−1^ (figure 1C). LED strips were mounted on an aluminium frame covered with a layer of half-white photographic LEE filters to diffuse the LED light without altering the spectrum and powered by a RIGOL DP711 programmable DC supply (RIGOL Technologies, Inc., Beaverton, USA). Light intensity (in quanta) at the tethered butterfly position was measured using an LI‑1500 light meter with an LI‑190R terrestrial quantum sensor (LI‑COR Inc., USA). We measured the LED spectrum using an MK350N Premium handheld spectrometer (UPRtek Inc., Taiwan). All electrical equipment used in experiments, such as power supplies (for LED light and magnetic coils), was grounded similarly to the behavioural set-up and housed in the smaller room. Radio-frequency noise, which could theoretically affect magnetic sensing of red admirals, was measured for both indoor and outdoor experiments using an FMZB 1512 calibrated active loop antenna (Schwarzbeck Mess-Elektronik, Germany) and a RIGOL DSA815-TG spectrum analyser (RIGOL Technologies, USA; supplementary material, figure S1). All indoor tests were conducted between 10.00 and 17.30 local time (GMT+2).

#### 2.3.2. Outdoor experimental procedures and light conditions

Experiments under full-spectrum natural light conditions were conducted outdoors at an open experimental site with local landmarks (trees and buildings) not visible to butterflies inside the simulator. Butterflies were exposed to the undisturbed natural magnetic field (intensity 50 507 ± 48 nT (mean ± s.d.) and inclination 69.9 ± 0.6°). Red admirals were tested under clear skies (10–20% cloud cover; total light intensity: approximately 12.4 × 10¹⁹ photons s⁻¹ m⁻², 380–420 nm: 2.7 × 10¹³ photons s⁻¹ cm⁻² nm⁻¹) with direct visibility of the sun, calm to light winds (less than or equal to 1–2 m s⁻¹) and temperatures of 19–25°C. Temperature (inside the flight simulator) and wind/airflow speed were checked before each test using a custom Arduino-based weather station (see details in https://magbbb.com/openscience/smartlab/) and a portable anemometer (CEM DT-618, Shenzhen Everbest, China), respectively. All tests were performed between 10.00 and 17.30 local time (GMT+2), when the sun was visible from within the simulator (except during clock-shift experiments; see details below).

#### 2.3.3. Test preparations and common procedures for all experimental conditions

Before testing, butterflies captured on the same day were randomly assigned to groups corresponding to the experimental conditions specific to a season and year (see details in subsequent sections). This approach minimized the effects of the season's progress and/or possible population-specific differences in migratory direction or flight motivation on the results. Prior to attachment to the tethering stalks, each red admiral was chilled in a refrigerator for 10–15 min to immobilize it. A short (3 cm) tungsten rod (non-ferromagnetic) with a footplate fixed to a small piece of paper (a tethering stalk) was glued vertically to the butterfly's thorax using Bostik Evo glue. After this procedure, butterflies were fed and returned to a mesh cage to recover before testing.

At the experimental site, cages were placed in direct sunlight for at least 30 min before testing to allow butterflies to warm up and perceive orientation cues. For clock-shift treatments, the cages were covered with diffusing (milky) glass to block sun and clear-sky cues. For experiments conducted in the magnetic coils (see §2.4), a 1 h acclimation period was provided before testing, similar to previous studies on monarch butterflies [22]. During this acclimation, butterflies adapted to the specific magnetic, light and temperature conditions of the treatment.

During testing, each butterfly was carefully removed from the mesh cage by grasping the tethering stalk and mounted on a flight-simulator rod. Each test lasted 15 min and was included in the analysis only if (i) the butterfly flew continuously (greater than 5 min without interruption) and (ii) flapping was vigorous, with a large and symmetrical wingbeat, indicating a secure attachment and no interference from the stalk. Assessments of flight continuity were made visually by A.P. To minimize potential handling effects, butterflies were given a 1 min acclimation period in the flight simulator after tethering before video recording commenced. The flight simulator was rotated randomly 90° clockwise or counterclockwise between trials to avoid directional bias from the apparatus.

### 2.4. Experimental conditions and groups

#### 2.4.1. Experiments under full-spectrum and artificial light during spring migration 2024

To date, only one study, based on visual observations, has reported northward flight in free-flying red admirals during spring migration [52], and no laboratory-controlled experiments have been conducted in spring. To address this gap, we captured red admirals in May 2024 and randomly assigned them to two experimental conditions (May–early June 2024): (i) outdoors, under full-spectrum natural light with full access to the natural magnetic field (NMF) and solar cues **(Spring Sun group**), and (ii) indoors, under artificial LED illumination with access to the NMF (**Spring LED group**).

#### 2.4.2. Experiments under full-spectrum natural light during autumn migration 2023–2024

To identify which cues (solar or magnetic) red admirals use to establish population-specific migratory direction in autumn, we ran experiments under varying light and magnetic conditions in August–September 2023–2024. First, butterflies were tested outdoors with full access to the NMF and solar cues (**Autumn Sun group**, 2023–2024). Second, to assess whether individuals can orient using magnetic cues alone, tests were conducted outdoors with access to the NMF but without clear sky or solar cues (**Magnetic cue-only group**, 2024). To block solar cues (no view of sun or sky-related cues such as spectral composition or light-intensity gradients), the top of the simulator was covered with a UV-transmissive diffusing sheet (LEE diffusion gel filter). This set-up reduced light intensity (total: approx. 2.4 × 10¹⁹ photons s⁻¹ m⁻², 380–420 nm: 2.1 × 10¹³ photons s⁻¹ cm⁻² nm⁻¹) compared with clear-sky conditions but without altering the spectrum of natural light (figure 1C). Additionally, butterflies were tested outdoors in the magnetic coils (figure 1B), which produced a magnetic field (MF) with the horizontal component rotated 120° clockwise (120° CW rotated MF, **Rotated MF group,** 2023). The rotated MF (RMF) closely matched the natural magnetic field in intensity (NMF: 50 455 ± 145 nT; inclination: 70.1 ± 0.25°) but differed in declination (RMF: 119.8 ± 1.2°; total intensity: 50 554 ± 97 nT; inclination: 69.95 ± 0.6°). In these tests, butterflies had no access to solar cues because the top of the set-up was covered with a diffuser.

To investigate the time-dependence of the sun compass in red admirals, we placed butterflies in a windowless room with controlled temperature (21–22°C) and an artificial light regime (see §2.1). Butterflies were subjected to a 6 h delayed clock-shift relative to local daylight time (lights on 6 h after local sunrise and off 6 h after local sunset; **Clock-shift group**, 2024). They were kept under conditions matching the control sun experiments (identical mesh cages, constant temperature and controlled humidity) for at least 7 days before the first test. The sun's azimuth changes on average by approximately 15° per hour; therefore, if red admirals use a time-dependent sun compass, a 6 h delay should produce an approximately 90° clockwise shift in orientation relative to control. Because the sun's azimuthal rate varies with time of day and latitude, the actual 6 h azimuthal change at our site ranged from 92° to 104° (mean = 99°), according to the free software Stellarium. Flight-simulator experiments for the Clock-shift group followed the same protocol as the outdoor control tests, with trials starting at least 1 h after the artificial ‘sunrise’ (lights on). All tests were performed between 12.00 and 17.30 local time (GMT+2). Because of the 6 h delayed photoperiod, the butterflies’ internal clocks corresponded to morning/daytime during testing (i.e. their subjective day began 6 h later), ensuring experiments did not occur during their subjective night.

#### 2.4.3. Experiments under artificial LED light during autumn migration 2023–2024

To examine the role of magnetic cues in orientation, red admirals were tested indoors in a flight simulator placed inside the magnetic coils (supplementary material, figure S1D). Indoor testing under artificial light excluded natural solar cues, including polarized skylight, which we could not fully control in outdoor experiments as we lacked specialized tools to measure polarization under the diffuser. First, butterflies were tested under artificial UV+white LED illumination (peak approx. 395 nm) in the NMF to assess baseline orientation in the absence of natural solar cues (**Autumn LED group,** 2023–2024). Second, to test for an inclination compass similar to that of monarch butterflies, we reversed the inclination angle of the magnetic field using magnetic coils and tested individuals under this modified magnetic field (**Inverted MF group,** 2023). The magnetic coils generated an artificial magnetic field matching the NMF in total intensity but with the vertical component inverted (intensity: 50 470 ± 180 nT, inclination: −70.2 ± 0.13°).

Third, to test responses to a controlled visual cue, butterflies were exposed to a simulated sun stimulus (**Green LED groups**, 2024). A single green LED served as an artificial solar cue under NMF conditions, in combination with UV+white LED background illumination. Insects, such as monarch butterflies and fruit flies, can use the position of a single green diode as a visual cue (simulated sun), either orienting towards the simulated sun or maintaining arbitrary headings when they consistently maintain a fixed but non-specific direction relative to the position of the green cue [54–56]. Such cues can be used to create a cue conflict between visual and magnetic information or to investigate whether insects can establish directed flight when only magnetic cues are available, and no significant orientation is observed in the absence of a visual cue. We mounted three green LEDs (530 nm peak, approx. 3 × 10^17^ photons s⁻¹ m⁻²; see spectrum here [57]) on the inner wall at positions of 0°, 120° and 240° relative to magnetic North, creating a symmetrical array. Only one LED was illuminated during each trial. The inner wall was draped with a black curtain to eliminate reflections from the green LEDs, which could otherwise create light artefacts. One group was tested in the NMF with the green LED positioned at 0° (**0_GreenLED group**) and another group with the LED at 120° (**120_GreenLED group**). The background illumination intensity and spectrum were identical to those used in the indoor experiments. Butterflies in experiments with the green LED positioned at 0° and 120° were tested across comparable periods of the day, and the order of LED-position treatments was balanced as far as possible across experimental days.

### 2.5. Data acquisition and statistical analysis

To study the orientation of red admirals under different light and magnetic conditions, we used video recordings as the primary source of directional data. All videos were analysed with DeepLabCut, an open source markerless pose-estimation software [58], to extract the coordinates of the butterfly's body and head in each frame. Using these coordinates and custom Python scripts, we calculated each individual's mean flight direction (*α*) and *r* value (resultant vector length), which indicates the directedness of the tested butterfly (its tendency to fly in the same direction; *r* value ranges from 0 to 1).

The classical Rayleigh test of uniformity (RT) was used to compare the group mean orientation against uniformity [59]. In addition, we applied a second-order statistical approach, the non-parametric Moore's modified Rayleigh test (MMRT; [60]), which weights mean angles according to their *r* values. For both RT and MMRT, *p* < 0.05 indicates a significant preferred direction (modified version of R script from [21]). Differences in orientation between experimental groups were assessed with the non-parametric Mardia–Watson–Wheeler (MWW) test. We also performed a maximum likelihood analysis using the CircMLE R package [61] to classify orientation patterns under different experimental conditions.

If one of the maximum likelihood models indicated a homogeneous symmetrical bimodal distribution, we additionally analysed orientation using doubled-angle analysis [59]. Differences in *r* values among groups of red admirals were tested with the non-parametric Kruskal–Wallis test, and differences in the proportion of butterflies meeting inclusion criteria were tested with Fisher's exact test. We also used bootstrap to test whether oriented groups were significantly more directed than non-significantly oriented groups (see details in [57–62]). Bootstrap, maximum likelihood analyses, MMRT, Kruskal–Wallis and Fisher's tests were conducted in R 4.3.2 [63]. The MWW test was performed in Oriana 4.02 (Kovach Computing Services, UK). RT calculations and group orientation visualizations were generated using a custom Python script.

## 3. Results

Mean directions and vector lengths for all individual butterflies included in the final analysis are provided in supplementary material, table S1. Summary information for each experimental group—including the number of individuals tested and included in the final analysis, median individual resultant vector length and results of MMRT—is presented in table 1. Results of the maximum likelihood estimation are provided in table S2 in the supplementary material.

**Table 1.** Information for each experimental group tested under different light and magnetic conditions.

season/year	experimental group	*N* of all tested butterflies in each group	*N* of butterflies with flight >5 min	Median individual resultant vector length, *r*	MMRT
spring 2024	Spring Sun	57	25	0.45	axial: *α* = 169 and 349°, R* = 1.3, 0.005 < *p* < 0.01
	Spring LED	56	23	0.67	*α* = 194°, R* = 0.53, 0.1 < *p* < 0.5
autumn 2023–2024	Autumn Sun 2023–2024	48	28	0.44	*α* = 152°, R* = 1.62, *p* < 0.001, 95% CI = 126–181°
	Magnetic cue-only 2024	35	12	0.68	*α* = 70°, R* = 0.61, 0.1 < *p* < 0.5
	Rotated MF 2023	53	20	0.42	*α* = 107°, R* = 0.45, 0.5 < *p* < 0.9
	Autumn Sun 2024	28	16	0.42	*α* = 162°, R* = 1.29, 0.005 < *p* < 0.01, 95% CI = 121–191°
	+6 h Clock-shift 2024	42	21	0.48	*α* = 195°, R* = 1.05, 0.025 < *p* < 0.05, 95% CI = 148–240°
	Autumn LED 2023–2024	102	35	0.42	*α* = 18°, R* = 1.11, *p* < 0.05
	Inverted MF 2023	51	17	0.57	*α* = 53°, R* = 0.04, 0.99 < *p* < 0.995
	0_GreenLED 2024	32	18	0.55	*α* = 303°, R* = 1.022, 0.05 < *p* < 0.1
	120_GreenLED 2024	39	17	0.51	*α* = 45°, R* = 0.86, 0.1 < *p* < 0.5

### 3.1. Orientation under full-spectrum natural and artificial light during spring migration 2024

In spring, red admirals tested with full access to natural cues (Spring Sun group) exhibited axially bimodal orientation, with headings towards the north and south (RT: *α* = 171 and 351°, *r* = 0.43, *n* = 25, 95% CI = 152–190° and 332–10°, *p* = 0.01; figure 2A). Results from maximum likelihood estimation (figure 2A; supplementary material, table S2A) and analysis of doubled angles (RT_doubled_: *α* = 338°, *r* = 0.43, *n* = 25, *p* = 0.008) further supported this pattern.

**Figure 2.**
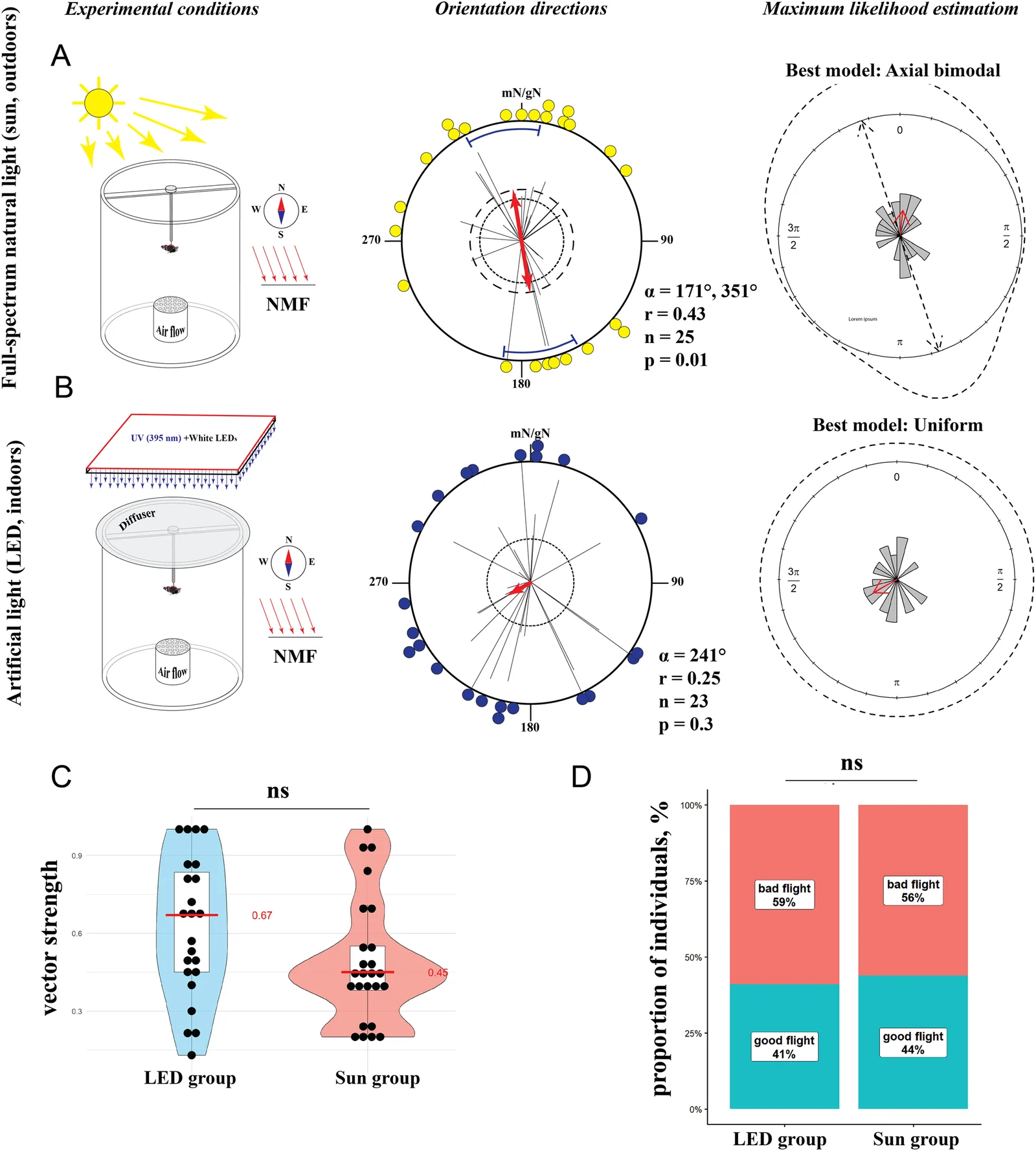
Orientation of red admirals under natural and artificial light conditions during spring migration 2024. (A) Spring Sun group: outdoors, natural light conditions and NMF. (B) Spring LED group: indoors, artificial LED light and NMF. Left side: Schematic illustration of the experimental set-up and experimental conditions. Middle: The flight directions of red admirals tested under full-spectrum (A) or LED (B) lights. Each dot at the circle periphery indicates the mean orientation of an individual butterfly. Dashed lines indicate the significance thresholds of the Rayleigh test for 5 and 1%, respectively. The mean direction of the admirals is shown as a red line; thin black lines are individual vectors of each red admiral; the curved lines indicate 95% confidence intervals. mN, magnetic North; gN, geographic North and NMF, the natural magnetic field. Right side: Results of maximum likelihood estimation. (C) The vector strength (directedness of a flight) in Spring LED and Spring Sun groups; each dot represents an individual *r* value, and a red line is the median *r* value for each group. (D) The proportion (%) of red admirals that flew continuously for more (‘good flight’) or less (‘bad flight’) than 5 min in each group.

In the absence of celestial cues (Spring LED group), red admirals showed random flight directions at the group level when the magnetic field was the only available directional cue (RT: *α* = 241°, *r* = 0.25, *n* = 23, *p* = 0.3; figure 2B). The median individual *r* value was higher in the Spring LED group (0.67) than in the Spring Sun group (0.45), and the difference in individual directedness between the groups was statistically significant, although relatively small (Kruskal–Wallis test: *χ*² = 3.96, *p* = 0.046; figure 2C). There was no difference between groups in the proportion of red admirals that flew continuously for more than 5 min and were included in the final analysis (Fisher's exact test: *p* = 0.85; figure 2D).

### 3.2. Orientation under full-spectrum natural light during autumn migration 2023–2024

When red admirals had full access to the natural sky and solar cues, together with an undisturbed magnetic field, they exhibited the seasonally appropriate migratory direction in the flight simulator: butterflies were oriented towards the south-southeast (RT: *α* = 169°, *r* = 0.59, *n* = 28, 95% CI = 144–193°, *p* < 0.001; figure 3A). Similar results were obtained in our previous study at the same location during autumn 2020–2021 [44], although butterflies in that study showed a more southwesterly mean direction (*α* = 198°). The difference in orientation between the two periods was not statistically significant (MWW test: *W* = 0.659, *p* = 0.719), and individual flight directedness did not differ between groups (Kruskal–Wallis test: *χ*² = 0.012, *p* = 0.91).

**Figure 3.**
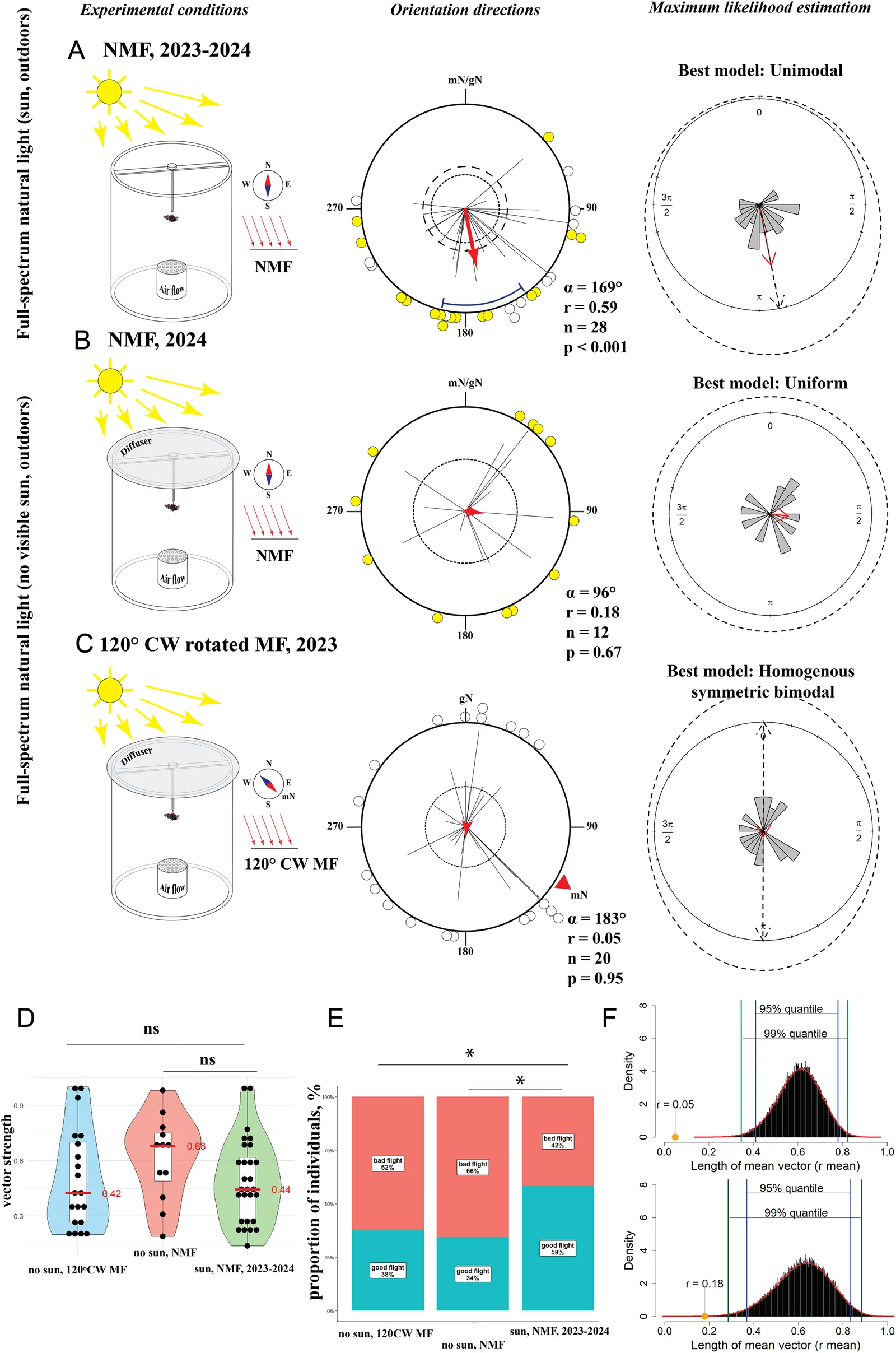
Orientation of red admirals under full-spectrum natural light during autumn migration 2023–2024. (A) Autumn Sun group: the natural sky, the sun and natural magnetic field (NMF), 2023: white dots, 2024: yellow dots. (B) Magnetic cue-only group: without access to solar cues, NMF, 2024. (C) Rotated MF group: without access to solar cues, 120° CW Rotated MF, 2023. For details, see legend in figure 1. (D) The vector strength (directedness of a flight) in Autumn Sun (sun, NMF, 2023–2024), Magnetic cue-only (no sun, NMF, 2024) and Rotated MF (no sun, 120 CW rotated MF) groups; each dot represents an individual *r* value, and a red line is the median *r* value for each group. (E) The proportion (%) of red admirals that flew continuously for more (‘good flight’) or less (‘bad flight’) than 5 min in each group. (F) The results of the bootstrap analysis for Magnetic cue-only (up) and Rotated MF (down) groups. The orange dot is the mean vector length in each group.

In the absence of solar cues, red admirals were unable to determine an appropriate population-specific migratory direction at the group level. Butterflies from the Rotated MF and Magnetic cue-only groups exhibited predominantly random flight direction at the group level, although some individuals maintained directed flight (see below). When the upper part of the flight simulator was covered with a diffuser, the orientation of red admirals did not differ from a uniform distribution in either the natural magnetic field (RT: *α* = 96°, *r* = 0.18, *n* = 12, *p* = 0.67; figure 3B) or a 120° clockwise-rotated magnetic field (RT: *α* = 183°, *r* = 0.05, *n* = 20, *p* = 0.95; figure 3C). Maximum likelihood modelling indicates a symmetrical bimodal distribution in the Rotated MF group; however, this pattern was not supported by the doubled-angle analysis (RT_doubled_: *α* = 4°, *r* = 0.3, *p* = 0.17). There was no significant difference in directedness (*r* value) between butterflies tested with and without access to solar cues (Kruskal–Wallis test: Autumn Sun vs Magnetic cue-only, *χ*² = 2.45, *p* = 0.12; Autumn Sun vs Rotated MF, *χ*² = 0.04, *p* = 0.83; figure 3D), although the median *r* value was higher in the Magnetic cue-only group (0.68) than in the Autumn Sun (0.44) and Rotated MF groups (0.42). Absence of solar cues slightly reduced the proportion of butterflies exhibiting continuous flights longer than 5 min in both the overcast and rotated MF conditions compared with controls tested under a clear sky (Fisher's exact test: Autumn Sun vs Magnetic cue-only, *p* = 0.044; Autumn Sun vs Rotated MF, *p* = 0.047; figure 3E). Bootstrap analysis showed that directions under overcast conditions were significantly more scattered than under clear-sky conditions (Autumn Sun vs Magnetic cue-only: *p* < 0.01; 95% CI for *r* = 0.41–0.89; 99% CI for *r* = 0.32–0.93; Autumn Sun vs Rotated MF: *p* < 0.01; 95% CI for *r* = 0.41–0.78; 99% CI for *r* = 0.34–0.83; figure 3F).

Animals that rely on solar cues typically compensate for changes in solar azimuth over the course of the day and, therefore, respond to clock-shift manipulations. However, after being maintained for at least one week under a 6 h delayed artificial photoperiod, red admirals tested during autumn 2024 still exhibited a southward mean direction (RT: *α* = 174°, *r* = 0.45, *n* = 21, *p* = 0.015, 95% CI = 136–216°; figure 4A), similar to that of non-shifted controls (RT: *α* = 179°, *r* = 0.65, *n* = 16, *p* = 0.001, 95% CI = 148–204°; figure 4B). Orientation did not differ between clock-shifted and control groups (MWW test: *W* = 3.56, *p* = 0.17), nor did flight directedness (Kruskal–Wallis test: *χ*² = 1.99, *p* = 0.15; figure 4C) or the proportion of individuals flying continuously for more than 5 min (Fisher's exact test: *p* = 0.63; figure 4D).

**Figure 4.**
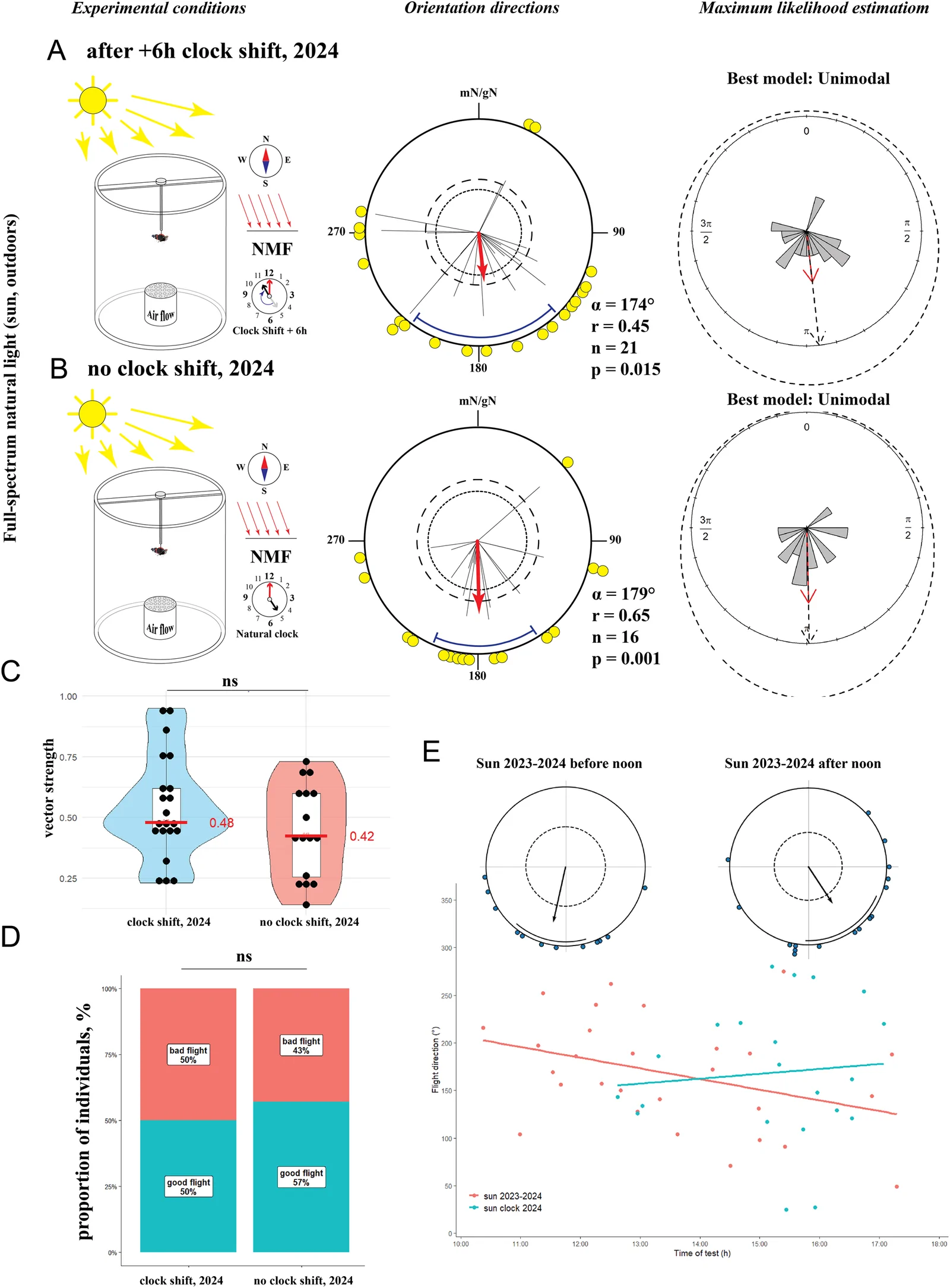
Orientation of red admirals after a 6-h delayed clock-shift during autumn migration 2024. (A) Clock-shift group: the natural sky, the Sun and NMF; (B) Autumn Sun 2024 group: the natural sky, the Sun and NMF. For details, see legend in figure 1. (C) The vector strength (directedness of a flight) in the Autumn Sun 2024 and Clock-shift groups; each dot represents an individual *r* value, and a red line is the median *r* value for each group. (D) The proportion (%) of red admirals that flew continuously for more (‘good flight’) or less (‘bad flight’) than 5 min in each group. (E) The orientation angles of red admirals tested with access to a clear sky are plotted as a function of the experimental time. Linear regressions of the particular datasets of angles are represented by the red (Autumn Sun group) and blue (Clock-shift group) lines. Diagrams above represent the orientation of red admirals from the Autumn Sun group before and after noon.

### 3.3. Orientation under artificial light during autumn migration 2023–2024

A more complex pattern emerged in indoor experiments, where red admirals were tested under artificial LED lighting in the NMF during autumn 2023–2024. According to the RT, butterflies displayed orientation indistinguishable from random when exposed solely to magnetic cues under artificial light (*α* = 7°, *r* = 0.20, *n* = 35, *p* = 0.25; figure 5A). However, likelihood-based modelling (figure 5A) and analysis of doubled angles (RT_doubled_: *α* = 26°, *r* = 0.29, *n* = 35, *p* = 0.051) suggested that the distribution may be close to bimodal.

**Figure 5.**
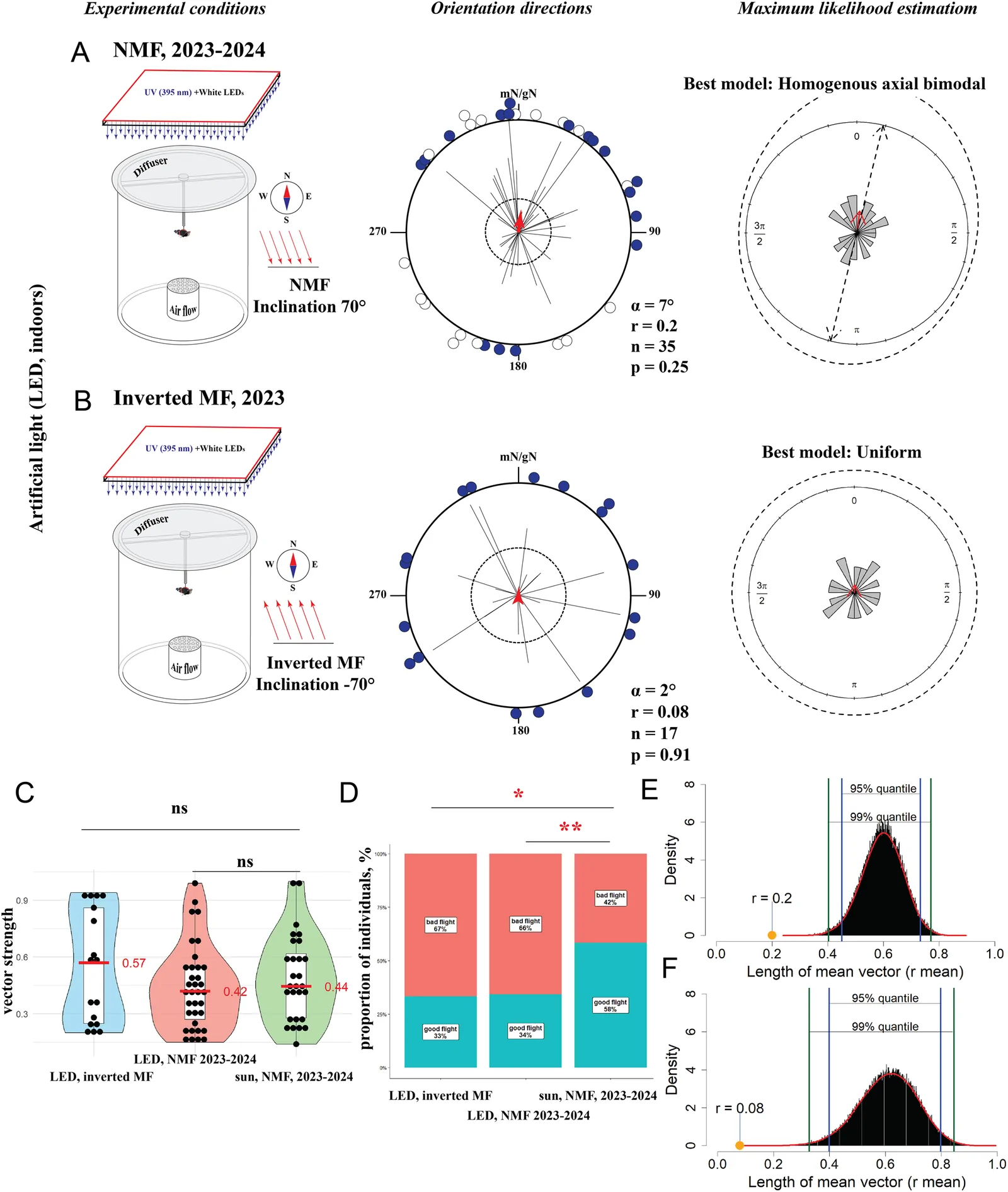
Orientation of red admirals under artificial LED light during autumn migration 2023–2024. (A) Autumn LED group: natural magnetic field (NMF), 2023: white dots, 2024: blue dots; (B) Inverted MF group: MF with inverted inclination, 2023. For details, see legend in figure 1. (C) The vector strength (directedness of a flight) in Autumn Sun (sun, NMF, 2023–2024), Autumn LED (LED, NMF, 2023–2024) and Inverted MF (LED, Inverted MF, 2023) groups; each dot represents an individual *r* value, and a red line is the median *r* value for each group. (D) The proportion (%) of red admirals that flew continuously for more (‘good flight’) or less (‘bad flight’) than 5 min in each group. (E) The results of the bootstrap analysis for Autumn LED group. The orange dot is the mean vector length. (F) The results of the bootstrap analysis for Inverted MF group. The orange dot is the mean vector length.

When analysed separately, the 2 years showed different responses. In 2023, butterflies tended to orient towards the northeast (RT: *α* = 28°, *r* = 0.40, *n* = 17, *p* = 0.065; MMRT: *α* = 33°, R* = 1.04, *n* = 17, 0.025 < *p* < 0.05; supplementary material, figure S2). In contrast, in 2024, red admirals exhibited random flight behaviour as a group under the same conditions (RT: *α* = 292°, *r* = 0.14, *n* = 18, *p* = 0.695; MMRT: *α* = 342°, R* = 0.54, *n* = 18, 0.1 < *p* < 0.5; supplementary material, figure S2), as did individuals tested under identical lighting but with reversed magnetic inclination (−70° MF) during autumn 2023 (RT: *α* = 2°, *r* = 0.08, *n* = 17, *p* = 0.91; figure 5B). Individual flight directedness did not differ significantly between experiments conducted under full-spectrum natural light outdoors and artificial LED conditions (Kruskal–Wallis test: Autumn Sun vs Autumn LED, *χ*² = 1.07, *p* = 0.29; Autumn Sun vs Inverted MF, *χ*² = 0.35, *p* = 0.55; figure 5C). However, the absence of solar cues under artificial light markedly reduced the proportion of butterflies that flew continuously for more than 5 min compared with outdoor tests (Fisher's exact test: Autumn Sun vs Autumn LED, *p* = 0.008; Autumn Sun vs Inverted MF, *p* = 0.016; figure 5D). Bootstrap analysis comparing butterflies tested under natural sunlight with those tested under artificial light revealed a significant difference (*p* < 0.01), with 95% confidence intervals for *r* ranging from 0.45 to 0.73 and 99% confidence intervals from 0.40 to 0.77 (figure 5E). Bootstrap analysis of the Inverted MF condition further confirmed the loss of orientation (*p* < 0.01; 95% CI: 0.34–0.78; 99% CI: 0.28–0.84; figure 5F).

The addition of a visual cue (a green LED) improved orientation under indoor conditions. Butterflies in the 0_GreenLED group did not orient directly towards the LED (the 95% CI did not include the diode position; RT: *α* = 306°, *r* = 0.46, *n* = 18, *p* = 0.022, 95% CI = 267–350°; figure 6A). Instead, most individuals maintained the LED on the right side of their visual field. When the LED position was shifted 120° clockwise (from magnetic north to southeast; 120_GreenLED group), butterflies showed a corresponding shift in orientation (RT: *α* = 60°, *r* = 0.51, *n* = 17, *p* = 0.01, 95% CI = 15–92°; figure 6B). Although the difference in orientation between the groups was not statistically significant (MWW test: *W* = 5.361, *p* = 0.07), it approached the conventional significance threshold (*α* = 0.05). The non-overlapping 95% (and 99%) confidence intervals nevertheless indicate a potential difference in orientation between the groups. There were no differences in directedness (vector length) between butterflies tested outdoors under natural sunlight and those tested with a simulated sun (Kruskal–Wallis test: Autumn Sun vs 0_GreenLED, *χ*² = 0.26, *p* = 0.61; Autumn Sun vs 120_GreenLED, *χ*² = 0.11, *p* = 0.74; figure 6C), nor in the proportion of individuals flying continuously for more than 5 min (Fisher's exact test: Autumn Sun vs 0_GreenLED, *p* = 0.20; Autumn Sun vs 120_GreenLED, *p* = 1.00; figure 6D). Similar to the Clock-shift group when headings were expressed relative to the sun's position, butterflies in both green LED treatments maintained a broadly similar angular relationship to the visual cue, generally keeping the LED on the right side of their visual field (figure 6E).

**Figure 6.**
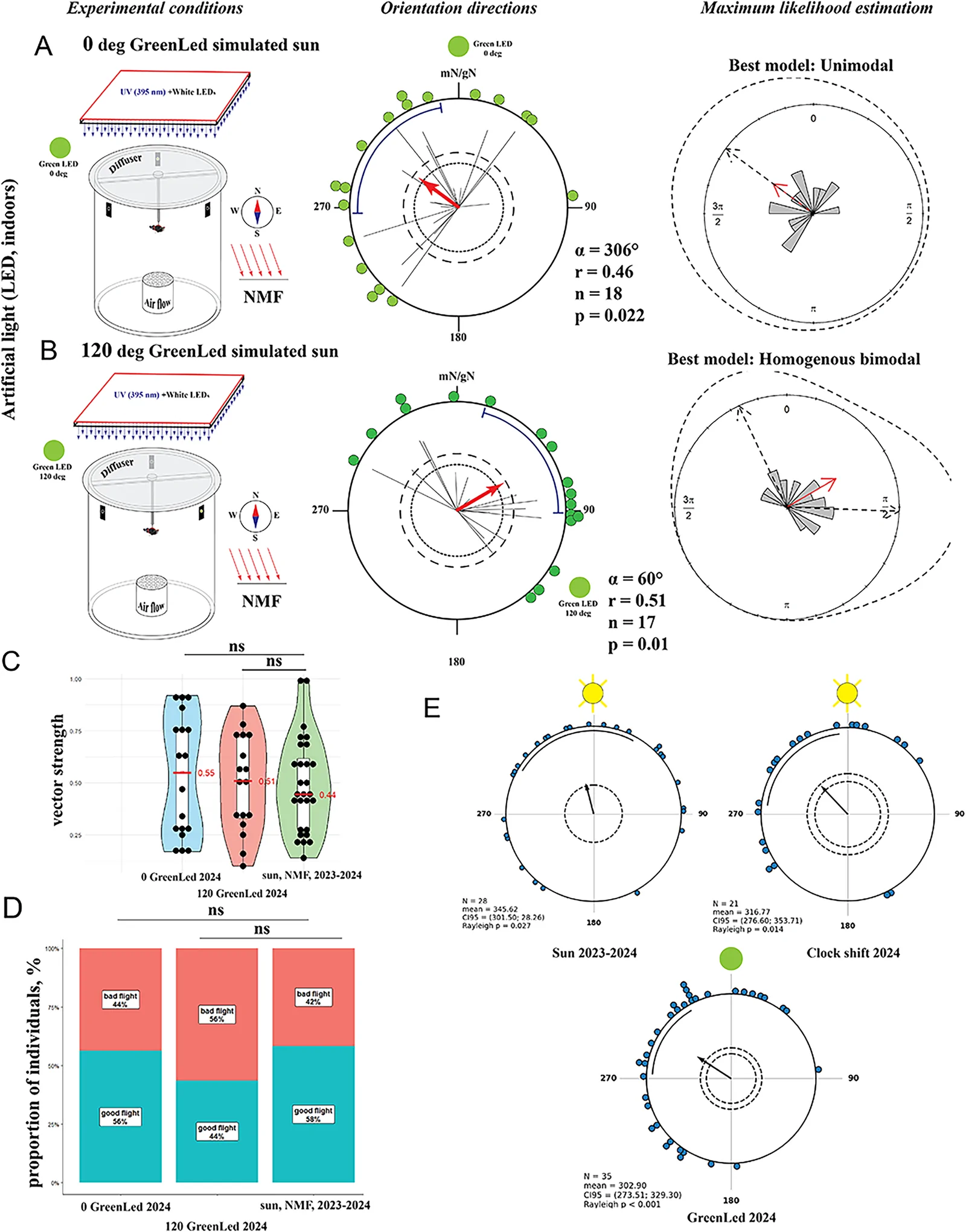
Orientation of red admirals under artificial LED light conditions with a green LED as a simulated sun during autumn migration 2024. (A) 0_GreenLED group: NMF, a green LED positioned at 0° (mN); (B) 120_GreenLED group: NMF, a green LED positioned at 120° (mSE); for details, see legend in figure 1. (C) The vector strength (directedness of a flight) in Autumn Sun (sun, NMF, 2023–2024), 0_GreenLED (2024) and 120_GreenLED (2024) groups; each dot represents an individual *r* value, and a red line is the median *r* value for each group. (D) The proportion (%) of red admirals that flew continuously for more (‘good flight’) or less (‘bad flight’) than 5 min in each group. (E) Orientation of red admirals relative to the position of the sun (upper diagrams) or green LED (lower diagram), with the cue position defined as 0°.

## 4. Discussion

### 4.1. Orientation of red admirals with full access to solar cues and the natural magnetic field

Red admiral autumn flights in Europe span southeast to southwest, based on vanishing-direction studies of free-flying butterflies [41,48–52,64,65] (see figure 1 and [44] for details). Little is known about their spring migration direction; however, one study based on visual observations in the wild reported predominantly northward flights in southern Europe during spring [52]. Directional data from over 40 years of red admiral trapping with Rybachy-type large traps on the eastern Baltic coast show predominantly southward movements in autumn, while spring captures were evenly split between opposite-facing traps, indicating bidirectional migration in this region (supplementary material, figure S1; see [44] for details). This pattern is similar to that observed in free-flying migratory bats further north along the Baltic coastline during autumn migration [66]. Earlier laboratory tests using circular arenas and video recording showed that red admirals can orient towards the southern migratory direction during autumn [47]. We later confirmed these results with a flight-simulator set-up [44], now regarded as a gold standard for investigating orientation mechanisms in migratory insects [67]. In the present study, red admirals tested in the flight simulator under sunny conditions in autumn preferred a south-southeast direction. During spring migration, individuals tested under the natural magnetic field and a clear sky exhibited an axially bimodal orientation pattern. Although unexpected, this pattern could result from a mixture of northward-moving migrants and local or relatively sedentary butterflies flying in the opposite direction at our field site, as suggested by the long-term trapping data (supplementary material, figure S1).

Bimodal orientation is common in migrating animals and has been documented in free-flying and laboratory-tested birds and bats [66,68,69]; however, its causes remain poorly understood. The observed variation may reflect heterogeneity in population origin, generation, migratory state, destination or movement strategy. Wild-caught populations may contain a continuum of movement phenotypes, from relatively sedentary individuals and dispersers to active seasonal migrants, potentially influenced by developmental conditions such as photoperiod and temperature. Such heterogeneity could contribute both to the opposing spring headings and to variation between southeasterly and southwesterly autumn headings, although the autumn distributions did not differ statistically between study periods. Because we did not measure morphology, physiology, genetics, developmental history or migratory state, these explanations remain hypotheses requiring targeted investigation. Despite the more complex spring behaviour, our study is the first to demonstrate that tethered red admirals in a flight simulator show seasonally appropriate, directed flight in both spring and autumn migration. These results imply that individuals may use solar cues (sun azimuth, polarized light, spectral contrast or light-intensity gradients) and/or the geomagnetic field to maintain migratory direction, but the current experimental design cannot determine which cues are actually used.

### 4.2. Orientation of red admirals without access to solar cues under natural and artificial light conditions

When access to celestial cues was restricted—either by covering the flight simulator with a diffuser outdoors or by testing individuals indoors under artificial light—so that the geomagnetic field remained the only available directional cue, red admirals were disoriented at the group level. The Autumn LED group was completely disoriented at the group level in 2024 but showed a tendency to orient in a non-migratory northeastern direction in 2023 (supplementary material, figure S2). Pooled across both years, directional preferences under these conditions tended to be bidirectional at the group level. Similar atypical non-migratory orientation under artificial light has been reported for migratory songbirds tested under narrowband (e.g. green, UV and blue) light at intensities higher than natural conditions or in total darkness. Under such lighting, animals often fail to show the expected migratory direction and instead exhibit altered (so-called fixed-direction responses) or bidirectional orientation patterns ([53,70]; see supplementary material, table S1 in [57] for examples). Therefore, it is possible that the unnatural lighting conditions in our experiments induced a comparable effect in red admirals.

Analysis of long-term phenology data from migratory traps along the eastern Baltic coast between 1983 and 2022 [44] shows that red admiral abundance is strongly associated with clear skies. Of 20 672 individuals recorded, 44.2% were captured under clear skies (0–25% cloud cover), 47.8% under partially cloudy conditions (25–50% cloud cover), 8% when cloud cover exceeded 75%, including only 1% under total overcast. Visual observations from other parts of Europe support this pattern: red admiral migration typically occurs on warm, clear, sunny days with favourable winds [48,65], while overcast skies and/or strong winds tend to suppress migratory activity. By contrast, North American monarchs appear less affected by overcast conditions, with flight motivation and bearings showing little change under cloudy skies [71].

The lack of group-level orientation when celestial cues were not visible may reflect a non-specific effect on flight motivation rather than orientation ability *per se*: red admirals may be less inclined to fly without a visible sky. In our experiments, restricting the direct view of the sky significantly reduced the number of individuals that exhibited continuous flight in the flight simulator (figures 3E and 5D) compared with red admirals tested under a clear sky; it did not reduce individual flight directedness (vector length) for groups tested with and without celestial cues (figures 3D and 5C). Introducing a green LED as a simulated sun (Green LED groups) restored group-level directional flight relative to non-visual cue conditions (Autumn LED and Inverted MF groups) and increased the proportion of butterflies exhibiting continuous flights longer than 5 min to levels comparable with the Autumn Sun group (figure 6D). Unlike previous studies in which insects oriented towards a simulated sun or adopted a fixed, non-specific direction relative to it [54,56,72], red admirals typically kept the green LED on the right side of their visual field while flying in the flight simulator. However, they did not adopt a migratory direction under these conditions, and their orientation was more scattered than that of individuals tested with a clear sky (figure 6; see MMRT results in table 1). These results suggest the green LED was not perceived as a true sun cue by red admirals and that it may need to be combined with other celestial cues (e.g. polarized light, spectral composition or intensity gradients) to support accurate migratory orientation.

### 4.3. Orientation mechanisms in European red admirals during seasonal migrations

As diurnal migrants, red admirals predominantly rely on solar cues to select and maintain seasonal migratory headings, as monarchs do. However, beyond these features, these species differ markedly. Monarchs use a sun compass that requires a direct view of the sun: when the sun's apparent position is displaced (e.g. shifted to the opposite hemisphere using a mirror) or replaced by a simulated green LED under a clear sky, monarchs follow the relocated cue and adjust their migratory direction accordingly [73], in contrast to red admirals [74]. Monarch butterflies can also use the sky's polarization pattern as a directional cue [73,75] (but see [76]) and perform time-compensated orientation as the sun moves across the sky [19,20], as many other diurnal navigators do (birds, hoverflies and fish) [5,8,21,77]. In contrast, red admirals failed to show the expected response to clock-shift experiments, consistent with previous studies on this species [47] and on painted ladies [39]. This does not imply that red admirals use a fixed angle relative to the sun's azimuth and maintain it throughout the day, as would be expected for a time-independent compass, and our results do not support such a mechanism (figure 4E). Instead, red admirals may rely on other skylight cues (spectral composition, polarized light, intensity gradients or combinations of these cues), without referencing a specific position of the sun in the sky, or on non-solar cues such as the geomagnetic field, at least at the individual level. Alternative explanations for these results are that the clock-shifting treatment may not have fully reset their internal clock [39], or that butterflies may have recalibrated their clock immediately before testing by retrieving time information from any number of sky-compass cues, such as solar elevation or the polarization pattern. Distinguishing among these assumptions will require further experiments under natural sky and tightly controlled laboratory conditions, including targeted manipulations of simulated sun cues (green light), polarized light and spectral composition.

Insects have experimentally demonstrated geomagnetic orientation across several taxa, including butterflies, moths and ants [22,28,78–81]. Unlike birds, which appear to use a light-dependent inclination magnetic compass [82], insects employ diverse magnetic mechanisms. Nocturnal migratory Lepidoptera (e.g. bogong moths and fall armyworms) use geomagnetic cues only in combination with visual cues, and their magnetic compass does not function in total darkness [28,81]. Ant magnetic sensing relies on the polarity of the Earth's magnetic field rather than its inclination [78]. Monarch butterflies possess an inclination-based magnetic compass that requires light of specific wavelengths and intensities to function properly [22,23]. Our results indicate that red admirals did not show evidence of a functional magnetic compass, as they were disoriented under both natural (indoors and outdoors) and altered magnetic field conditions when visual cues were absent, similar to findings reported for painted lady butterflies [39]. Some studies reporting no magnetic orientation in monarchs [20,76,83] may reflect insufficient illumination in the wavelength range required for their magnetic compass [22]; however, in our experiments, all light sources (natural and artificial) supplied sufficient intensity in the 380–420 nm range (figure 1C; §2). Thus, insufficient light is an unlikely explanation in our case, although we cannot exclude the possibility that red admirals’ magnetic sensitivity has different spectral or intensity thresholds than monarchs.

Red admirals’ magnetic orientation may require visual cues (landmarks or celestial patterns) to function properly, as seen in some nocturnal Lepidoptera [28,81], so the absence of such cues in the flight simulator could disrupt magnetic responses. Alternatively, only a subset of individuals may be capable of magnetic sensing or responsive to magnetic manipulations; this would result in disorientation at the group level while allowing directional behaviour in some individuals, as reported for non-migratory monarchs [84]. All these possibilities warrant further targeted experiments.

## 5. Conclusion

Although there are hundreds, if not thousands, of migratory lepidopteran species worldwide, most remain understudied, and only a small proportion has been investigated in detail, with a strong research bias towards monarch butterflies [85]. Despite the extensive understanding of orientation mechanisms in monarchs, these findings cannot be readily extrapolated to other migratory butterflies. Because monarchs have a unique migratory strategy—one generation migrates in autumn, overwinters in a restricted area and then resumes migration in spring—they may have evolved a more complex navigation system combining magnetic and celestial compasses [86]. By contrast, species such as red admirals and painted ladies have multigenerational migratory cycles without diapause, with different generations completing different parts of the annual migration to broad overwintering regions in Africa or the Mediterranean [33,38,47].

Maintaining multiple, energetically costly sensory systems and specialized compass circuitry in the central brain for precise navigation is probably favoured only when necessary for survival and reproduction [86]. It is, therefore, not surprising that insects requiring precise navigation over long or short distances, such as migratory monarch butterflies and bogong moths, as well as homing *Cataglyphis* ants [87,88], have evolved both celestial and magnetic orientation mechanisms. Consequently, species that do not require highly precise navigation to successfully reach their destinations during the annual cycle, such as some European migratory butterflies, may not employ multiple complex compass mechanisms to select and maintain their seasonal migratory direction but instead rely on simpler orientation strategies. The limited existing studies already reveal a diversity of orientation mechanisms among migratory Lepidoptera. Therefore, expanding research across species with different migratory strategies is essential to improve understanding of their navigation and how anthropogenic changes may negatively impact these systems.

## Supplementary Material



## Data Availability

All data, video analysis scripts, 3D printing and laser-cutting files, and Python and R scripts used for statistical analyses are publicly available in the Zenodo repository [89]. Supplementary material is available online [90].
