# Supplementary material for: Not all butterflies are monarchs: orientation using daytime celestial cues but not magnetic cues in European migratory red admirals (Vanessa atalanta)
Source: J R Soc Interface. 2026 Oct 21;23(243):20260537. doi: 10.1098/rsif.2026.0537 (PMC13682934; doi:10.1098/rsif.2026.0537)
Supplement: Supplementary file 1 [file rsif.2026.0537.zip › rsif.2026.0537/rsif20260537_si_001.docx]

**Supplementary materials**

**Not All Butterflies Are Monarchs: Orientation Using Daytime Celestial Cues but Not Magnetic Cues in European Migratory Red Admirals (*Vanessa atalanta*)**

**Figure S1. Experimental setups and the large ‘Rybachy-like’ traps for catching butterflies.**

**A)** Directional preferences of movements of red admirals (up: proportions of red admirals captured in each trap (NE and SW traps) from 1982 to 2022; down: photo of traps from a drone)

| **A** |  |
| --- | --- |
|  |  |
|  |  |
|  |  |

**Figure S1 (continue): B-C) A photo (B) and scheme (C) of the modified version of Mouritsen-Frost flight simulator.** Black boxes (2) indicate the positions of the miniature cameras used to record butterfly orientation in the flight simulator. The cameras were positioned above the arena around the tungsten rod to which the red admirals were tethered. **D) Experimental setup for indoor experiments. E) Spectra of SunLike lamps and ZooDa Bird lamps (SunLike lamps:** [**https://mlighting.jiagle.com/news/1171923.html**](https://mlighting.jiagle.com/news/1171923.html)**; ZooDA Bird Lamp:** [**https://zooda.ru/product/лампа-для-птиц-zooda-bird-lamp-е27-2-4-uvb-12-uva/**](https://zooda.ru/product/лампа-для-птиц-zooda-bird-lamp-е27-2-4-uvb-12-uva/) **; F) Average amplitude of the magnetic field noise measured at the outdoor experimental site as a function of the central position of the 10 kHz detection window**. 1 – near the box with minicomputer Raspberry PI (2 m away from the flight simulator), 2 - inside the flight simulator, the place where red admiral was attached to a vertical encoder shaft; all electronic equipment was turned on, 3 - inside the flight simulator, the place where red admiral was attached to a vertical encoder shaft; all electronic equipment was turned off.

| **B** | **C** | **D** |
| --- | --- | --- |
|  |  |  |
| **E** | | **F** |
|  | |  |

| **Figure S1 (continue).** Average amplitude of the magnetic field noise measured at the laboratory chamber as a function of the central position of the 10 kHz detection window. A – all electronic equipment OFF, B – all electronic equipment ON, ∆ f = 10 kHz | | |
| --- | --- | --- |
| **A** |  |  |
| **B** |  |  |

**Table S1. Mean directions and vector lengths of all red admirals included in the final analysis. Bold and blue numbers are 2024 data.**

| **Year** | **Season** | **№ of active admirals** | **Magnetic condition** | **Light source** | **Mean direction, α** | **Length of mean vector, r** |
| --- | --- | --- | --- | --- | --- | --- |
| **2024** | **Spring** | 1 | **Natural magnetic field** | **Full-spectrum light (outdoors, sun)** | 287 | 0.55 |
|  |  | 2 |  |  | 275 | 0.39 |
|  |  | 3 |  |  | 310 | 0.2 |
|  |  | 4 |  |  | 327 | 0.71 |
|  |  | 5 |  |  | 328 | 0.68 |
|  |  | 6 |  |  | 12 | 0.2 |
|  |  | 7 |  |  | 334 | 0.84 |
|  |  | 8 |  |  | 161 | 0.49 |
|  |  | 9 |  |  | 173 | 0.4 |
|  |  | 10 |  |  | 167 | 0.93 |
|  |  | 11 |  |  | 131 | 0.24 |
|  |  | 12 |  |  | 249 | 0.46 |
|  |  | 13 |  |  | 0 | 0.38 |
|  |  | 14 |  |  | 12 | 0.47 |
|  |  | 15 |  |  | 148 | 0.45 |
|  |  | 16 |  |  | 166 | 0.93 |
|  |  | 17 |  |  | 131 | 0.2 |
|  |  | 18 |  |  | 19 | 0.2 |
|  |  | 19 |  |  | 352 | 0.41 |
|  |  | 20 |  |  | 5 | 0.43 |
|  |  | 21 |  |  | 187 | 1 |
|  |  | 22 |  |  | 44 | 0.46 |
|  |  | 23 |  |  | 20 | 0.24 |
|  |  | 24 |  |  | 22 | 0.54 |
|  |  | 25 |  |  | 56 | 0.4 |
| **2024** | **Spring** | 1 | **Natural magnetic field** | **Full-spectrum light (indoors, UV + White LEDs)** | 60 | 0.67 |
|  |  | 2 |  |  | 125 | 1 |
|  |  | 3 |  |  | 356 | 1 |
|  |  | 4 |  |  | 124 | 0.68 |
|  |  | 5 |  |  | 203 | 0.85 |
|  |  | 6 |  |  | 313 | 0.13 |
|  |  | 7 |  |  | 193 | 0.82 |
|  |  | 8 |  |  | 187 | 0.46 |
|  |  | 9 |  |  | 155 | 1 |
|  |  | 10 |  |  | 260 | 0.2 |
|  |  | 1 |  |  | 209 | 1 |
|  |  | 12 |  |  | 333 | 0.3 |
|  |  | 13 |  |  | 153 | 0.8 |
|  |  | 14 |  |  | 15 | 0.88 |
|  |  | 15 |  |  | 331 | 0.53 |
|  |  | 16 |  |  | 4 | 0.4 |
|  |  | 17 |  |  | 226 | 0.68 |
|  |  | 18 |  |  | 4 | 0.57 |
|  |  | 19 |  |  | 192 | 0.44 |
|  |  | 20 |  |  | 242 | 0.5 |
|  |  | 21 |  |  | 298 | 0.72 |
|  |  | 22 |  |  | 245 | 0.23 |
|  |  | 23 |  |  | 240 | 0.49 |
| **2024** | **Autumn** | 1 | **Natural magnetic field** | **Full-spectrum light (outdoors, sun)** | 104 | 0.6 |
|  |  | 2 |  |  | 197 | 0.26 |
|  |  | 3 |  |  | 186 | 0.21 |
|  |  | 4 |  |  | 216 | 0.4 |
|  |  | 5 |  |  | 169 | 0.6 |
|  |  | 6 |  |  | 213 | 0.14 |
|  |  | 7 |  |  | 194 | 0.5 |
|  |  | 8 |  |  | 104 | 0.41 |
|  |  | 9 |  |  | 252 | 0.24 |
|  |  | 10 |  |  | 262 | 0.42 |
|  |  | 11 |  |  | 189 | 0.21 |
|  |  | 12 |  |  | 141 | 0.6 |
|  |  | 13 |  |  | 172 | 0.7 |
|  |  | 14 |  |  | 144 | 0.43 |
|  |  | 15 |  |  | 188 | 0.67 |
|  |  | 16 |  |  | 49 | 0.73 |
| **2024** | **Autumn** | 1 | **Natural magnetic field** | **Full-spectrum light (outdoors, sun), Clock Shift +6h** | 126 | 0.45 |
|  |  | 2 |  |  | 186 | 0.48 |
|  |  | 3 |  |  | 201 | 0.62 |
|  |  | 4 |  |  | 27 | 0.52 |
|  |  | 5 |  |  | 129 | 0.75 |
|  |  | 6 |  |  | 143 | 0.24 |
|  |  | 7 |  |  | 134 | 0.32 |
|  |  | 8 |  |  | 117 | 0.58 |
|  |  | 9 |  |  | 25 | 0.47 |
|  |  | 10 |  |  | 109 | 0.45 |
|  |  | 11 |  |  | 254 | 0.48 |
|  |  | 12 |  |  | 220 | 0.95 |
|  |  | 13 |  |  | 280 | 0.93 |
|  |  | 14 |  |  | 271 | 0.86 |
|  |  | 15 |  |  | 269 | 0.46 |
|  |  | 16 |  |  | 121 | 0.23 |
|  |  | 17 |  |  | 219 | 0.58 |
|  |  | 18 |  |  | 221 | 0.25 |
|  |  | 19 |  |  | 177 | 0.76 |
|  |  | 20 |  |  | 148 | 0.62 |
|  |  | 21 |  |  | 162 | 0.43 |
| **2024** | **Autumn** | **1** | **Natural magnetic field** | **Full-spectrum light (indoors, UV + White LEDs)/ Green LED as a simulated sun (position 0 deg)** | 238 | 0.28 |
|  |  | 2 |  |  | 223 | 0.34 |
|  |  | 3 |  |  | 8 | 0.17 |
|  |  | 4 |  |  | 37 | 0.86 |
|  |  | 5 |  |  | 253 | 0.92 |
|  |  | 6 |  |  | 321 | 0.92 |
|  |  | 7 |  |  | 345 | 0.18 |
|  |  | 8 |  |  | 271 | 0.17 |
|  |  | 9 |  |  | 21 | 0.75 |
|  |  | 10 |  |  | 280 | 0.25 |
|  |  | 11 |  |  | 85 | 0.28 |
|  |  | 12 |  |  | 216 | 0.9 |
|  |  | 13 |  |  | 332 | 0.63 |
|  |  | 14 |  |  | 279 | 0.28 |
|  |  | 15 |  |  | 334 | 0.76 |
|  |  | 16 |  |  | 302 | 0.63 |
|  |  | 17 |  |  | 225 | 0.75 |
|  |  | 18 |  |  | 40 | 0.47 |
| **2024** | **Autumn** | 1 | **Natural magnetic field** | **Full-spectrum light (indoors, UV + White LEDs)/ Green LED as a simulated sun (position 120 deg)** | 47 | 0.16 |
|  |  | 2 |  |  | 17 | 0.33 |
|  |  | 3 |  |  | 122 | 0.51 |
|  |  | 4 |  |  | 332 | 0.74 |
|  |  | 5 |  |  | 334 | 0.72 |
|  |  | 6 |  |  | 140 | 0.5 |
|  |  | 7 |  |  | 75 | 0.56 |
|  |  | 8 |  |  | 93 | 0.3 |
|  |  | 9 |  |  | 92 | 0.1 |
|  |  | 10 |  |  | 358 | 0.36 |
|  |  | 11 |  |  | 95 | 0.78 |
|  |  | 12 |  |  | 296 | 0.87 |
|  |  | 13 |  |  | 82 | 0.74 |
|  |  | 14 |  |  | 55 | 0.25 |
|  |  | 15 |  |  | 135 | 0.36 |
|  |  | 16 |  |  | 88 | 0.63 |
|  |  | 17 |  |  | 312 | 0.57 |
| **2023-2024** | **Autumn** | 1 | **Natural magnetic field** | **Full-spectrum light (outdoors, sun)** | 240 | 0.44 |
|  |  | 2 |  |  | 128 | 0.98 |
|  |  | 3 |  |  | 71 | 0.22 |
|  |  | 4 |  |  | 150 | 0.28 |
|  |  | 5 |  |  | 156 | 0.27 |
|  |  | 6 |  |  | 157 | 0.45 |
|  |  | 7 |  |  | 239 | 0.44 |
|  |  | 8 |  |  | 189 | 0.58 |
|  |  | 9 |  |  | 98 | 1 |
|  |  | 10 |  |  | 275 | 0.5 |
|  |  | 11 |  |  | 91 | 0.71 |
|  |  | 12 |  |  | 131 | 0.77 |
|  |  | 13 |  |  | **104** | **0.6** |
|  |  | 14 |  |  | **197** | **0.26** |
|  |  | 15 |  |  | **186** | **0.21** |
|  |  | 16 |  |  | **216** | **0.4** |
|  |  | 17 |  |  | **169** | **0.6** |
|  |  | 18 |  |  | **213** | **0.14** |
|  |  | 19 |  |  | **194** | **0.5** |
|  |  | 20 |  |  | **104** | **0.41** |
|  |  | 21 |  |  | **252** | **0.24** |
|  |  | 22 |  |  | **262** | **0.42** |
|  |  | 23 |  |  | **189** | **0.21** |
|  |  | 24 |  |  | **141** | **0.6** |
|  |  | 25 |  |  | **172** | **0.7** |
|  |  | 26 |  |  | **144** | **0.43** |
|  |  | 27 |  |  | **188** | **0.67** |
|  |  | 28 |  |  | **49** | **0.73** |
| **2023-2024** | **Autumn** | 1 | **Natural magnetic field** | **Full-spectrum light (indoors, UV + White LEDs)** | **341** | **0.6** |
|  |  | 2 |  |  | **161** | **0.5** |
|  |  | 3 |  |  | **30** | **0.47** |
|  |  | 4 |  |  | **9** | **0.18** |
|  |  | 5 |  |  | **358** | **0.55** |
|  |  | 6 |  |  | **335** | **0.53** |
|  |  | 7 |  |  | **337** | **0.55** |
|  |  | 8 |  |  | **22** | **0.4** |
|  |  | 9 |  |  | **199** | **0.18** |
|  |  | 10 |  |  | **211** | **0.17** |
|  |  | 11 |  |  | **236** | **0.2** |
|  |  | 12 |  |  | **209** | **0.34** |
|  |  | 13 |  |  | **255** | **0.15** |
|  |  | 14 |  |  | **231** | **0.21** |
|  |  | 15 |  |  | **130** | **0.37** |
|  |  | 16 |  |  | **156** | **0.7** |
|  |  | 17 |  |  | **310** | **0.84** |
|  |  | 18 |  |  | **68** | **0.34** |
|  |  | 19 |  |  | 36 | 0.99 |
|  |  | 20 |  |  | 355 | 0.84 |
|  |  | 21 |  |  | 355 | 0.29 |
|  |  | 22 |  |  | 47 | 0.25 |
|  |  | 23 |  |  | 309 | 0.32 |
|  |  | 24 |  |  | 190 | 0.4 |
|  |  | 25 |  |  | 67 | 0.44 |
|  |  | 26 |  |  | 40 | 0.21 |
|  |  | 27 |  |  | 82 | 0.42 |
|  |  | 28 |  |  | 25 | 0.89 |
|  |  | 29 |  |  | 304 | 0.22 |
|  |  | 30 |  |  | 96 | 0.47 |
|  |  | 31 |  |  | 181 | 0.3 |
|  |  | 32 |  |  | 69 | 0.56 |
|  |  | 33 |  |  | 325 | 0.43 |
|  |  | 34 |  |  | 197 | 0.48 |
|  |  | 35 |  |  | 352 | 0.67 |
| **2024** | **Autumn** | 1 | **Natural magnetic field** | **Full-spectrum light (outdoors, no sun, diffuser)** | 42 | 0.67 |
|  |  | 2 |  |  | 30 | 0.74 |
|  |  | 3 |  |  | 196 | 0.19 |
|  |  | 4 |  |  | 159 | 0.52 |
|  |  | 5 |  |  | 155 | 0.55 |
|  |  | 6 |  |  | 275 | 0.4 |
|  |  | 7 |  |  | 244 | 0.69 |
|  |  | 8 |  |  | 125 | 0.78 |
|  |  | 9 |  |  | 52 | 0.31 |
|  |  | 10 |  |  | 38 | 0.98 |
|  |  | 11 |  |  | 94 | 0.86 |
|  |  | 12 |  |  | 303 | 0.7 |
| **2023** | **Autumn** | **1** | **Artificial magnetic field (-70 deg inclination)** | **Full-spectrum light (indoors, UV + White LEDs)** | 255 | 0.24 |
|  |  | 2 |  |  | 288 | 0.57 |
|  |  | 3 |  |  | 7 | 0.6 |
|  |  | 4 |  |  | 45 | 0.25 |
|  |  | 5 |  |  | 336 | 0.86 |
|  |  | 6 |  |  | 237 | 0.92 |
|  |  | 7 |  |  | 110 | 0.2 |
|  |  | 8 |  |  | 285 | 0.21 |
|  |  | 9 |  |  | 75 | 0.79 |
|  |  | 10 |  |  | 101 | 0.92 |
|  |  | 11 |  |  | 44 | 0.28 |
|  |  | 12 |  |  | 22 | 0.2 |
|  |  | 13 |  |  | 332 | 0.91 |
|  |  | 14 |  |  | 181 | 0.35 |
|  |  | 15 |  |  | 145 | 0.94 |
|  |  | 16 |  |  | 238 | 0.37 |
|  |  | 17 |  |  | 172 | 0.61 |
| **2023** | **Autumn** | 1 | **120˚ CW Rotated magnetic field** | **Full-spectrum light (outdoors, no sun - diffusor)** | 188 | 0.27 |
|  |  | 2 |  |  | 134 | 1 |
|  |  | 3 |  |  | 186 | 0.73 |
|  |  | 4 |  |  | 304 | 0.36 |
|  |  | 5 |  |  | 209 | 0.21 |
|  |  | 6 |  |  | 42 | 0.52 |
|  |  | 7 |  |  | 8 | 0.94 |
|  |  | 8 |  |  | 144 | 0.74 |
|  |  | 9 |  |  | 134 | 0.98 |
|  |  | 10 |  |  | 135 | 0.69 |
|  |  | 11 |  |  | 31 | 0.36 |
|  |  | 12 |  |  | 23 | 0.41 |
|  |  | 13 |  |  | 147 | 0.26 |
|  |  | 14 |  |  | 224 | 0.2 |
|  |  | 15 |  |  | 236 | 0.2 |
|  |  | 16 |  |  | 346 | 0.57 |
|  |  | 17 |  |  | 8 | 0.34 |
|  |  | 18 |  |  | 284 | 0.2 |
|  |  | 19 |  |  | 244 | 0.62 |
|  |  | 20 |  |  | 0 | 0.44 |

**Table S2. The results of maximum likelihood estimation for all experimental groups.**

The best-fitted models are listed in the corresponding column (model names are from Fitak & Johnsen, 2017)

*** -** Best model to describe distribution, **bold –** other probable models to describe distribution (deltaAICc < 2)

**A) Sun/NMF, outdoor, spring 2024**

| Model | Description | deltaAICc | AIC_weights |
| --- | --- | --- | --- |
| **M4B** | **Axial bimodal** | **0.000*** | **0.31*** |
| M5B | Bimodal | 2.38 | 0.17 |
| **M4A** | **Homogenous axial bimodal** | ***0.743*** | ***0.14*** |
| **M3A** | **Homogenous symmetric bimodal** | ***0.156*** | ***0.14*** |
| **M3B** | **Symmetric bimodal** | ***0.757*** | ***0.07*** |
| M5A | Homogenous bimodal | 3.224 | 0.06 |
| M2B | Symmetric modified unimodal | 4.110 | 0.02 |
| M2C | Modified unimodal | 5.432 | 0.013 |
| M1 | Uniform | 5.023 | 0.009 |
| M2A | Unimodal | 7.312 | 0.003 |

**B) Sun/NMF, outdoor, autumn 2023-2024**

| Model | Description | deltaAICc | AIC_weights |
| --- | --- | --- | --- |
| **M2A** | **Unimodal** | **0.000*** | **0.61*** |
| M5A | Homogenous bimodal | 3.523 | 0.19 |
| M5B | Bimodal | 6.426 | 0.08 |
| M2C | Modified unimodal | 4.833 | 0.07 |
| M2B | Symmetric modified unimodal | 5.617 | 0.04 |
| M3B | Symmetric bimodal | 8.292 | 0.012 |
| M4B | Axial bimodal | 13.044 | 0.0017 |
| M4A | Homogenous axial bimodal | 13.658 | 0.0009 |
| M1 | Uniform | 16.804 | 0.00011 |
| M3A | Homogenous symmetric bimodal | 21.075 | 0.00002 |

**C) No_Sun(diffusor)/NMF, outdoor, autumn 2024**

| Model | Description | deltaAICc | AIC_weights |
| --- | --- | --- | --- |
| **M1** | **Uniform** | **0.000*** | **0.35*** |
| M3B | Symmetric bimodal | 4.163 | 0.2 |
| M4B | Axial bimodal | 7.084 | 0.18 |
| M2B | Symmetric modified unimodal | 4.535 | 0.07 |
| M2A | Unimodal | 4.539 | 0.07 |
| M3A | Homogenous symmetric bimodal | 4.931 | 0.057 |
| M2C | Modified unimodal | 7.578 | 0.035 |
| M4A | Homogenous axial bimodal | 8.040 | 0.028 |
| M5A | Homogenous bimodal | 12.624 | 0.01 |
| M5B | Bimodal | 19.348 | 0.003 |

**D) UV-White LED/NMF, indoor, spring 2024**

| Model | Description | deltaAICc | AIC_weights |
| --- | --- | --- | --- |
| **M1** | **Uniform** | **0*** | **0.23*** |
| **M3A** | **Homogenous symmetric bimodal** | **1.457** | **0.15** |
| M2A | Unimodal | 2.141 | 0.11 |
| M5A | Homogenous bimodal | 3.83 | 0.10 |
| M2B | Symmetric modified unimodal | 2.307 | 0.09 |
| M4A | Homogenous axial bimodal | 3.227 | 0.08 |
| M3B | Symmetric bimodal | 3.821 | 0.06 |
| M5B | Bimodal | 6.35 | 0.05 |
| M2C | Modified unimodal | 4.391 | 0.04 |
| M4B | Axial bimodal | 5.755 | 0.04 |

**E) UV-White LED/NMF, indoor, autumn 2023-2024**

| Model | Description | deltaAICc | AIC_weights |
| --- | --- | --- | --- |
| **M4A** | **Homogenous axial bimodal** | **0.000*** | **0.25*** |
| **M3A** | **Homogenous symmetric bimodal** | **0.359** | **0.17** |
| **M4B** | **Axial bimodal** | **1.673** | **0.15** |
| **M3B** | **Symmetric bimodal** | **1.822** | **0.01** |
| M5A | Homogenous bimodal | 2.500 | 0.096 |
| M5B | Bimodal | 4.304 | 0.056 |
| M2B | Symmetric modified unimodal | 2.668 | 0.055 |
| M1 | Uniform | 2.418 | 0.052 |
| M2C | Modified unimodal | 3.888 | 0.036 |
| M2A | Unimodal | 3.970 | 0.028 |

**F) UV-White LED/-70 deg CMF, indoor, autumn 2023**

| Model | Description | deltaAICc | AIC_weights |
| --- | --- | --- | --- |
| **M1** | **Uniform** | **0.000*** | **0.61*** |
| M2A | Unimodal | 4.660 | 0.09 |
| M2B | Symmetric modified unimodal | 4.667 | 0.09 |
| M3A | Homogenous symmetric bimodal | 4.713 | 0.088 |
| M3B | Symmetric bimodal | 7.644 | 0.033 |
| M4A | Homogenous axial bimodal | 7.644 | 0.033 |
| M2C | Modified unimodal | 7.888 | 0.029 |
| M5A | Homogenous bimodal | 10.979 | 0.013 |
| M4B | Axial bimodal | 11.698 | 0.009 |
| M5B | Bimodal | 15.608 | 0.004 |

**G) GreenLed_North/NMF, indoor, autumn 2024**

| Model | Description | deltaAICc | AIC_weights |
| --- | --- | --- | --- |
| **M2A** | **Unimodal** | **0.000*** | **0.43*** |
| M2B | Symmetric modified unimodal | 2.292 | 0.14 |
| M5A | Homogenous bimodal | 2.969 | 0.098 |
| M5B | Bimodal | 3.025 | 0.0955 |
| M2C | Modified unimodal | 3.029 | 0.00953 |
| M1 | Uniform | 4.061 | 0.056 |
| M3B | Symmetric bimodal | 5.636 | 0.026 |
| M4A | Homogenous axial bimodal | 5.734 | 0.025 |
| M4B | Axial bimodal | 6.124 | 0.02 |
| M3A | Homogenous symmetric bimodal | 7.170 | 0.01 |

**H) GreenLed_SouthEast/NMF, indoor, autumn 2024**

| Model | Description | deltaAICc | AIC_weights |
| --- | --- | --- | --- |
| **M5A** | **Homogenous bimodal** | **0.000*** | **0.57*** |
| M5B | Bimodal | 4.074 | 0.21 |
| **M2A** | **Unimodal** | **1.164** | **0.09** |
| M2B | Symmetric modified unimodal | 2.172 | 0.06 |
| M2C | Modified unimodal | 5.081 | 0.021 |
| M3B | Symmetric bimodal | 5.241 | 0.02 |
| M4A | Homogenous axial bimodal | 6.719 | 0.009 |
| M4B | Axial bimodal | 8.631 | 0.007 |
| M1 | Uniform | 5.643 | 0.006 |
| M3A | Homogenous symmetric bimodal | 6.853 | 0.005 |

**J) Sun/NMF, outdoor, autumn 2024**

| Model | Description | deltaAICc | AIC_weights |
| --- | --- | --- | --- |
| **M2A** | **Unimodal** | **0.000*** | **0.48*** |
| M2B | Symmetric modified unimodal | 2.259 | 0.16 |
| M5A | Homogenous bimodal | 5.518 | 0.12 |
| M2C | Modified unimodal | 3.901 | 0.12 |
| M3B | Symmetric bimodal | 5.417 | 0.06 |
| M5B | Bimodal | 9.673 | 0.05 |
| M4A | Homogenous axial bimodal | 7.758 | 0.02 |
| M4B | Axial bimodal | 13.556 | 0.0021 |
| M1 | Uniform | 10.458 | 0.002 |
| M3A | Homogenous symmetric bimodal | 13.042 | 0.0007 |

**K) Sun/NMF, Clock shift +6h, outdoor, autumn 2024**

| Model | Description | deltaAICc | AIC_weights |
| --- | --- | --- | --- |
| **M2A** | **Unimodal** | **0.000*** | **0.31*** |
| M5B | Bimodal | 3.733 | 0.26 |
| **M2B** | **Symmetric modified unimodal** | **1.402** | **0.16** |
| M2C | Modified unimodal | 3.196 | 0.09 |
| M5A | Homogenous bimodal | 4.791 | 0.07 |
| M3B | Symmetric bimodal | 5.062 | 0.04 |
| M1 | Uniform | 4.206 | 0.027 |
| M4B | Axial bimodal | 7.060 | 0.023 |
| M4A | Homogenous axial bimodal | 6.055 | 0.022 |
| M3A | Homogenous symmetric bimodal | 8.442 | 0.005 |

**L) No Sun/120˚CW CMF, outdoors, autumn 2023**

| Model | Description | deltaAICc | AIC_weights |
| --- | --- | --- | --- |
| **M3A** | **Homogenous symmetric bimodal** | **0.486*** | **0.23*** |
| **M1** | **Uniform** | **0*** | **0.20*** |
| M4B | Axial bimodal | 3.035 | 0.17 |
| M3B | Symmetric bimodal | 2.307 | 0.14 |
| M4A | Homogenous axial bimodal | 3.041 | 0.09 |
| M5B | Bimodal | 6.648 | 0.06 |
| M5A | Homogenous bimodal | 6.207 | 0.03 |
| M2B | Symmetric modified unimodal | 4.548 | 0.03 |
| M2A | Unimodal | 4.596 | 0.03 |
| M2C | Modified unimodal | 7.274 | 0.01 |

**Figure S2.** Results of the Rayleigh test (RT) and Moore’s Modified Rayleigh Test (MMRT) for the Autumn 2023–2024 group, analysed separately for 2023 and 2024.

|  | **MMRT** | **RT** |
| --- | --- | --- |
| **2023:**  **MMRT**  α = 33˚  n = 17  R* = 1.04  **0.025 < p < 0.05** |  |  |
| **2024:**  **MMRT**  α = 342˚  n = 18  R* = 0.54  0.1 < p < 0.5 |  |  |

**Figure S3. Virtual tracks of red admirals tested under different experimental conditions**

| **Spring Sun group** |  |  |
| --- | --- | --- |
| **Autumn Sun group** |  |  |
| **Autumn LED group** |  |  |
| **Invert MF group** |  |  |
| **Clock-shift group** |  |  |
| **GreenLed group** |  |  |
|  |  |  |
|  |  |  |
